# Metformin mitigates osteoarthritis progression by modulating the PI3K/AKT/mTOR signaling pathway and enhancing chondrocyte autophagy

**DOI:** 10.1515/biol-2022-0922

**Published:** 2024-07-29

**Authors:** Tianjie Xu, Kainan Liu, Jiaxin Fan, Xiang Jia, Xiaoling Guo, Xingwang Zhao, Yanhua Cao, Hui Zhang, Qian Wang

**Affiliations:** School of Basic Medical Sciences, North China University of Science and Technology, Tangshan, Hebei, 063000, China; Hebei Key Laboratory for Chronic Diseases, Tangshan, Hebei, 063000, China; Department of Basic Medicine, Xingtai Medical College, Xingtai, Hebei, 054000, China; Department of Orthopedics, Affiliated Hospital of North China University of Science and Technology, Tangshan, Hebei, 063000, China; School of Public Health, North China University of Science and Technology, Tangshan, Hebei, 063000, China; Department of Joint Surgery 1, The Second Hospital of Tangshan, Tangshan, Hebei, 063000, China

**Keywords:** Met, osteoarthritis, autophagy, cartilage, PI3K/AKT/mTOR

## Abstract

Osteoarthritis (OA) is a chronic degenerative disease characterized by overall joint tissue damage. Metformin (Met) has been shown to inhibit inflammatory reactions, though its potential protective mechanism on cartilage remains unclear. This study investigated Met’s potential to protect cartilage in an OA rat model. Various morphological experiments were conducted to assess changes in cartilage tissue morphology before and after Met treatment. Protein and mRNA levels of cartilage-specific genes were measured using western blot, immunohistochemical staining, and RT-qPCR. Additionally, protein levels of autophagy-related and mTOR pathway-related proteins were measured. The results indicate an imbalance in the synthesis and degradation metabolism of chondrocytes, downregulation of cellular autophagy, and activation of the PI3K/Akt/mTOR pathway after surgery. However, treatment with Met could upregulate the expression of synthetic metabolic factors, indicating its contribution to cartilage repair. Furthermore, analysis of autophagy and pathway protein levels indicated that Met effectively attenuated autophagic damage to osteoarthritic cartilage cells and abnormal activation of the PI3K/Akt/mTOR pathway. In conclusion, Met can inhibit the abnormal activation of the PI3K/AKT/mTOR signaling pathway in cartilage tissue, promote the restoration of cartilage cell autophagic function, improve the balance of cartilage cell synthesis and degradation metabolism, and thus exert a protective effect on rat joint cartilage.

## Graphical abstract

**Figure:**
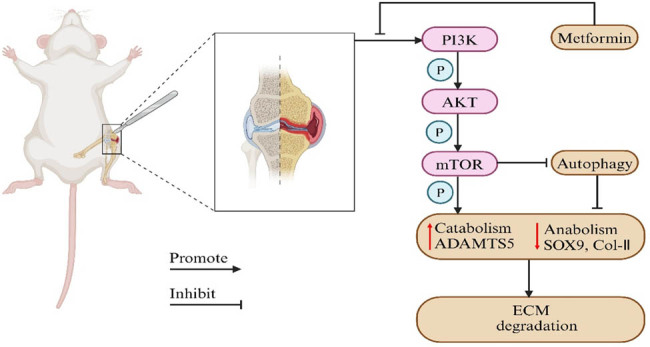

## 1 Introduction

Osteoarthritis (OA) is a chronic degenerative disease characterized by joint pain, functional impairment, and joint degeneration [1]. With over 500 million people worldwide affected by OA, this number is expected to continue to rise due to the increasing aging population [2]. Traditional treatment methods, such as non-steroidal anti-inflammatory drugs (NSAIDs) and physical therapy, have limited efficacy for OA, and surgical interventions, like joint replacement, are considered only when other treatments have failed [3]. Given the high incidence of OA, the prolonged treatment duration, and the significant side effects of medications on the gastrointestinal and central nervous systems [4], it is essential to identify for suitable drugs and target mechanisms for the prevention and treatment of OA.

Articular cartilage is composed of chondrocytes and extracellular matrix (ECM), with chondrocytes being the only cell type within the cartilage. Although it is known that factors such as gender, age, trauma, and mechanical stimulation significantly contribute to the increasing incidence of OA, the precise mechanisms underlying its development remain incompletely understood [5]. Current research suggests that inflammation may be associated with the occurrence and progression of OA [6]. Inflammatory cytokines inhibit the synthetic metabolic activity of chondrocytes, downregulate the synthesis of major ECM components, and increase degradation metabolism, leading to ECM degradation in articular cartilage and ultimately inducing cartilage degeneration [7]. The inflammatory response is closely linked to the activity of the phosphoinositide 3-kinase/protein kinase B (PI3K/AKT) signaling pathway. It has been demonstrated that abnormal activation of the phosphoinositide 3-kinase/protein kinase B/mammalian target of rapamycin (PI3K/AKT/mTOR) signaling pathway can promote chondrocyte apoptosis, inhibit of cellular autophagy, and induce degradation of specific genes in chondrocytes, thereby promoting the occurrence and progression of OA [8].

Metformin (Met) is the first-line medication for treating type 2 diabetes. Apart from its blood sugar-lowering effects, Met also exhibits anti-inflammatory, anti-tumor, anti-aging, and cardiovascular protective properties [9]. Research suggests that Met may impede the progression of OA by activating the adenosine 5′-monophosphate (AMP)-activated protein kinase pathway, indicating its potential clinical use in early-stage OA treatment [10]. Additionally, Met has been reported to alleviate OA by inhibiting ferroptosis and cellular senescence in chondrocytes, positioning it as a promising therapeutic agent for OA treatment [11,12]. Therefore, this study aimed to investigate the protective effects and potential mechanisms of Met in the treatment of OA through the PI3K/AKT/mTOR pathway to enhance our understanding of its role in OA treatment and provide experimental evidence for potentially enhancing its clinical application.

## 2 Materials and methods

### 2.1 Establishment of the OA rat model

A total of 40 standard deviation (SD) rats were randomly divided into four groups (*n* = 10): control group, sham group, OA group, and Met group. The OA rat model was established as previously described [13]. Briefly, the rats were anesthetized by intraperitoneal injection of 1% sodium pentobarbital at a dose of 100 mg/kg, following which they were placed in the supine position on a surgical table, their right knee joint was shaved and sterilized with iodine before being covered with sterile surgical drapes. A longitudinal incision of approximately 3 cm was made along the inner side of the right knee, followed by a small incision along the patellar ligament to expose the joint cavity. The inner collateral ligament was severed, and the medial meniscus and anterior cruciate ligament were resected. The incision was subsequently sutured layer-by-layer. They were then given a continuous injection of 50,000 units of penicillin 3 days postoperatively to prevent infection. In the sham group, the joint cavity was exposed without resection of the medial collateral ligament, anterior cruciate ligament, and medial meniscus. The control group did not undergo any surgical procedure.


**Ethical approval:** The research related to animal use has been complied with all the relevant national regulations and institutional policies for the care and use of animals.

### 2.2 Experimental animal grouping

The rats were divided into four groups, and the control group, sham group, and OA group were administered 10 mL/kg/day of normal saline, while the Met group received a daily oral gavage of 200 mg/kg/day Met. After 8 weeks of continuous administration, the rats were anesthetized with pentobarbital, and blood samples were collected from the abdominal aorta, followed by euthanasia. In addition, serum samples and the right knee joints were collected for further experiments.

### 2.3 Quantitative real-time PCR

Total RNA was extracted from the cartilage tissue using Trizol solution and reverse transcribed into complementary DNA (cDNA) using a cDNA synthesis kit. Quantitative real-time PCR was performed using SYBR Premix, with β-actin as the internal reference gene. The relative expression levels of the target genes compared to the control group were calculated using the 2^−ΔΔCt^ method. The primer sequences for the target genes are presented in the table below.

**Table:** 

Gene names	Primer sequence
β-actin	Forward:
	5′-TCCTGAGCGCAAGTACTCTG-3′
	Reverse:
	5′-GCTCAGTAACAGTCCGCCTA-3′
ADAMTS-5	Forward:
	5′-TCGAGAACCACATCCGCCTG-3′
	Reverse:
	5′-TCGTAGTGCTCCTCATGGTC-3′
SOX9	Forward:
	5′-CTACTCCACCTTCACCTAC-3′
	Reverse:
	5′-TCTGTCACCATTGCTCTT-3′
Col-Ⅱ	Forward:
	5′-CTCCTGGTACTGATGGTCCC-3′
	Reverse:
	5′-CAACATCACCTCTGTCTCCC-3'

### 2.4 Hematoxylin and eosin (H&E) staining

The resected knee joint tissue was fixed in 4% paraformaldehyde solution, fixed for 3 days and decalcified using a 10% ethylene diamine tetraacetic acid solution, with the solution changed every 48 h. Decalcification was considered complete when there was no resistance during needle puncture of the bone tissue after 8 weeks of continuous decalcification. The samples were then embedded in paraffin and coronal sections with a thickness of 6 μm were cut. After deparaffinization using xylene and graded ethanol, the sections were washed three times with water, stained with hematoxylin for 2 min, rinsed with running water, and stained with eosin for 1 min. After dehydration with xylene, the sections were sealed with optical resin and observed under a microscope.

### 2.5 Safranin O-fast green staining and Mankin classification

After dewaxing and hydrating the sections, they were stained with Weigert’s stain for 5 min and then rinsed with water. The sections were quickly differentiated with an acidic differentiation solution, immersed in a fast green staining solution for 5 min, and then rinsed with a weak acid solution. Then, they were stained with a fuchsin staining solution for 3 min, underwent a gradient ethanol dehydration and xylene clearing process and sealed with optical resin. Histopathological evaluation was conducted using the Mankin scoring system to assess the histopathological changes in the cartilage tissue based on the structure (score 0: normal surface, score 1: surface irregularity, score 2: surface irregularity and vascular haze, score 3: cracks extending into the cartilage transition layer, score 4: cracks extending into the cartilage radiating layer, score 5: cracks extending into the cartilage calcification layer, score 6: complete structural damage), chondrocytes (score 0: normal cell count, score 1: diffuse increase in cell count, score 2: cluster of proliferating cells, score 3: reduced cell count), safranin O-fast green staining (score 0: normal staining, score 1: mildly decreased, score 2: moderately decreased, score 3: severely decreased, score 4: unpigmented), and tidemarks (score 0: complete, score 1: vascular invasion).

### 2.6 Toluidine blue staining

After routine dewaxing and hydration of the sections in water, they were immersed in a toluidine blue staining solution for 30 min. The sections were then rinsed with water and differentiated in 95% ethanol, followed by dehydration in absolute ethanol and clearing in xylene. Lastly, they were sealed with an optical resin for the observation of cellular morphology under a microscope.

### 2.7 Western blot

After collecting the cartilage tissue, the cartilage cells were lysed using RIPA lysis buffer combined with protease and phosphatase inhibitors. The cells were further lysed using an ultrasonic disruptor, and the lysate was centrifuged at 12,000 rpm for 15 min at 4°C in a high-speed centrifuge. The collected protein supernatant was mixed with sample loading buffer at a 4:1 ratio and boiled at 100°C for 5 min. The gel was prepared according to the fast SDS-PAGE gel preparation instructions, and the samples were loaded. After electrophoresis and transfer, the membrane was blocked with 10% skim milk and incubated overnight at 4°C with the following primary antibodies: SRY-box transcription factor 9 (SOX9) (1:1,000; ET1611-56, Hua Bio, China), recombinant disintegrin and metalloproteinase with thrombospondin 5 (ADAMTS5) (1:2,000; DF13268, Affinity), type II collagen (Col-II) (1:3,000, AF0135, Affinity), P62 (1:500, WL02385, China), Beclin1 (1:2,000; WL02508, China), PI3K (1:500; WL02240, China), phosphorylated phosphoinositide-3 kinase (p-PI3K) (1:1,000; T40116F, China), AKT (1:1,000; WL0003b, China), phosphorylated protein kinase B (p-AKT) (1:2000; AF0016, Affinity), mTOR (1:500; WL02477, China), phosphorylated mammalian target of rapamycin (p-mTOR) (1:500; WL03694, China) for overnight at 4°C. After washing with tris buffered saline with tween 20 (TBST), the membrane was incubated with goat anti-rabbit IgG (H + L) secondary antibody (1:10,000; BA1054, Boster Bio, China) at room temperature for 90 min. After three washes with TBST for 10 min each, an enhanced chemiluminescence detection reagent was added, the gel was exposed to an imaging system, and the results were analyzed using ImageJ software.

### 2.8 Immunohistochemistry

The pre-treatment procedures of the sections were consistent with H&E staining. After rinsing with phosphate-buffered saline, the sections were incubated in a 0.1% trypsin solution at 37°C in a CO_2_ incubator for 30 min for antigen retrieval. The sections were then blocked with a peroxidase-blocking solution at room temperature for 10 min. Following this, the sections were incubated overnight at 4°C with the following primary antibodies: SOX9 (1:200), ADAMTS5 (1:200), Col-II (1:200), P62 (1:400), Beclin1 (1:200), PI3K (1:400), p-PI3K (1:300), AKT (1:200), p-AKT (1:200), mTOR (1:200), p-mTOR (1:400, WLH3897, China), and incubated with a horseradish peroxidase-conjugated goat anti-rabbit IgG polymer at 37°C for 30 min. Visualization was performed using 3,3′-diaminobenzidine, and the nuclei were counterstained with hematoxylin. Cellular positive expression was observed under a microscope.

### 2.9 Micro-CT

After removing the surrounding excess muscle tissue from the fixed knee joint, the region of interest (ROI) in the rat knee joint was scanned using micro-CT (SkyScan1176, Bruker, Kontich, Belgium). The scanning parameters were set as follows: voltage 40 keV, current 270 μA, and resolution 9 μm³. The ROI was defined as the area between the subchondral bone plate and the growth plate beneath the tibial plateau, excluding cortical bone. The collected data were processed and analyzed using the corresponding software. The measured parameters within the ROI included: bone mineral density (BMD, mg/cm³), bone volume fraction (BV/TV, %), trabecular separation degree (Tb.Sp, mm), trabecular number (Tb.N, 1/mm), and trabecular thickness (Tb.Th, mm).

### 2.10 Statistical analysis

Statistical analysis was performed using SPSS 26.0. All data are presented as mean ± SD. Differences between groups were analyzed using one-way analysis of variance followed by least significant difference *post*-*hoc* multiple comparisons. A significance level of *P* < 0.05 was considered statistically significant.

## 3 Results

### 3.1 Met improves cartilage damage in rats with OA

**Figure 1:**
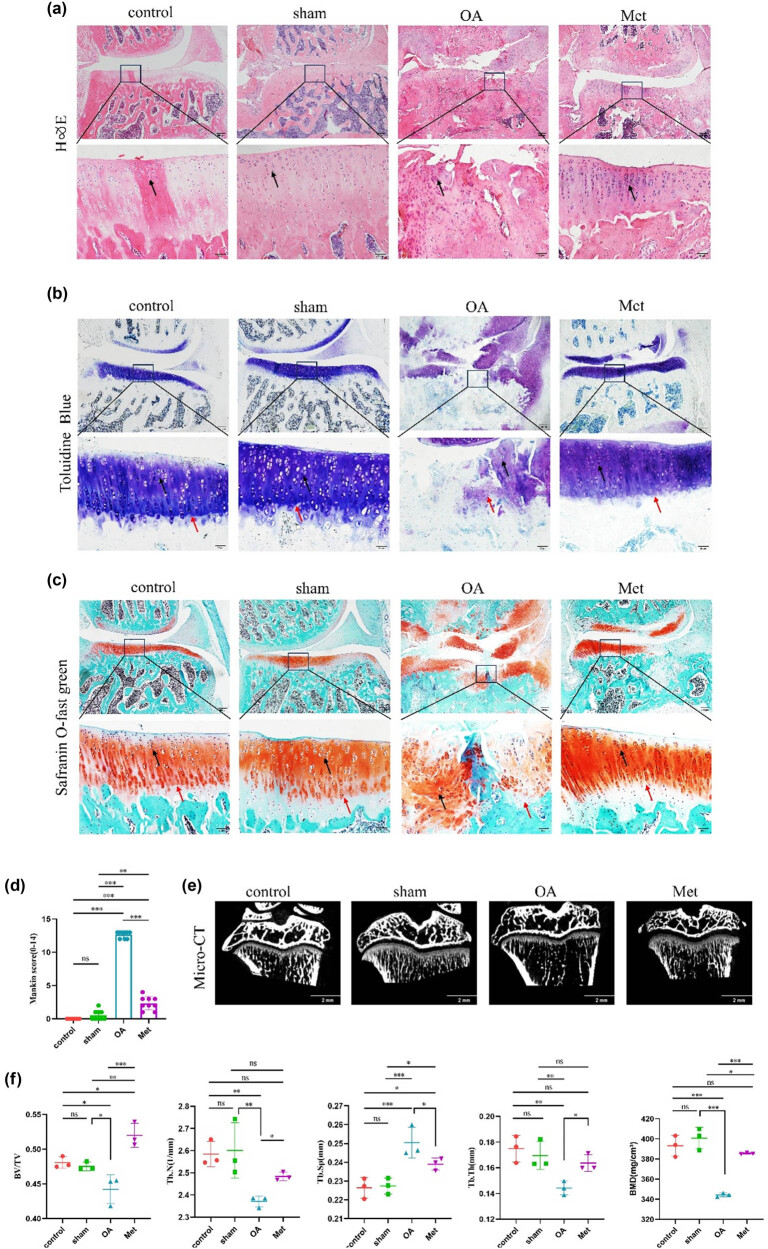
Met improves pathological damage in rats with OA. (a) H&E staining, (b) toluidine blue staining, (c) safranin O-fast green staining. Black arrows indicate chondrocytes and red arrows indicate to tidemarks. Scale bars = 200 and 50 μm. (d) Quantitative analysis of Mankin scores (*n* = 9). (e) Pathological changes in subchondral bone in articular cartilage detected by micro-CT scanning. Scale bar = 2 mm. (f) Quantitative analysis of parameters for subchondral bone remodeling in various groups of knee articular cartilage, including BV/TV, Tb.N, Tb.Sp, Tb.Th, and BMD. Data are presented as mean ± SD (*n* = 3). ns, no significance; *P < 0.05; **P < 0.01; ***P < 0.001.

To investigate the effects of Met on articular cartilage in OA rats, the degree of cartilage degeneration was assessed using H&E, toluidine blue, and safranin O-fast green staining. The H&E staining results revealed that the cartilage surface in the control group was smooth, exhibiting normal tissue morphology with singly arranged chondrocytes, showing no significant differences from the sham group. In contrast, the OA group displayed irregular cartilage surfaces, defects in cartilage tissue, a thinned cartilage layer, clustered chondrocytes, and a decreased cell number. Conversely, the Met group showed a significantly thickened cartilage layer, a smooth cartilage surface, increased cell numbers, and an orderly arrangement of chondrocytes (Figure 1a).

The toluidine blue staining results indicated that in the control and sham groups, the chondrocytes displayed blue-purple nuclei, with the cartilage matrix uniformly stained in blue or blue-purple, and intact tidemarks. However, in the OA group, the cartilage tissue exhibited uneven staining, with some areas remaining unstained and severe damage to the tidemark. Comparatively, the cartilage tissue in the Met group displayed uniform and deep staining, along with a higher number of chondrocytes and intact tidemarks (Figure 1b).

The safranin O-fast green staining results showed that in the control and sham groups, chondrocytes and the cartilage matrix were uniformly stained red, with intact tidemarks. In the OA group, cartilage tissue staining was uneven, tidemarks were severely damaged, and fast green staining was observed. In contrast, the Met group exhibited uniform and deep staining of cartilage tissue, along with intact tidemarks (Figure 1c). These findings were further validated using Mankin scores. In addition, we observed no significant difference in Mankin scores between the control and sham groups (*P* > 0.05), and compared to the control and sham groups, the OA group showed a significant increase in Mankin scores (*P* < 0.05), which were subsequently reduced by Met treatment (Figure 1d).

Additionally, micro-CT was performed to assess the subchondral bone microstructure in rat knee joints, and the results showed that the OA group had structural changes related to OA, including irregular joint surfaces, disordered arrangement of trabecular bone, and the formation of osteophytes, compared to the control and sham groups (Figure 1e). Comparatively, these osteoarthritic changes were less pronounced in the Met group, which exhibited a relatively smooth joint surface with fewer osteophytes and an increased number of trabeculae.

Furthermore, analysis of bone parameters such as BV/TV, BMD, Tb.Sp, Tb.N, and Tb.Th revealed no statistically significant differences between the sham and control groups (*P* > 0.05) (Figure 1f). Compared to the control and sham groups, the OA group exhibited decreased BV/TV, Tb.N, Tb.Th, and BMD, along with increased Tb.Sp (*P* < 0.05). Notably, compared to the OA group, the Met group showed increased BV/TV, Tb.N, Tb.Th, and BMD but decreased Tb.Sp (*P* < 0.05).

### 3.2 Met inhibits the catabolic metabolism in cartilage tissues of rats with OA

**Figure 2:**
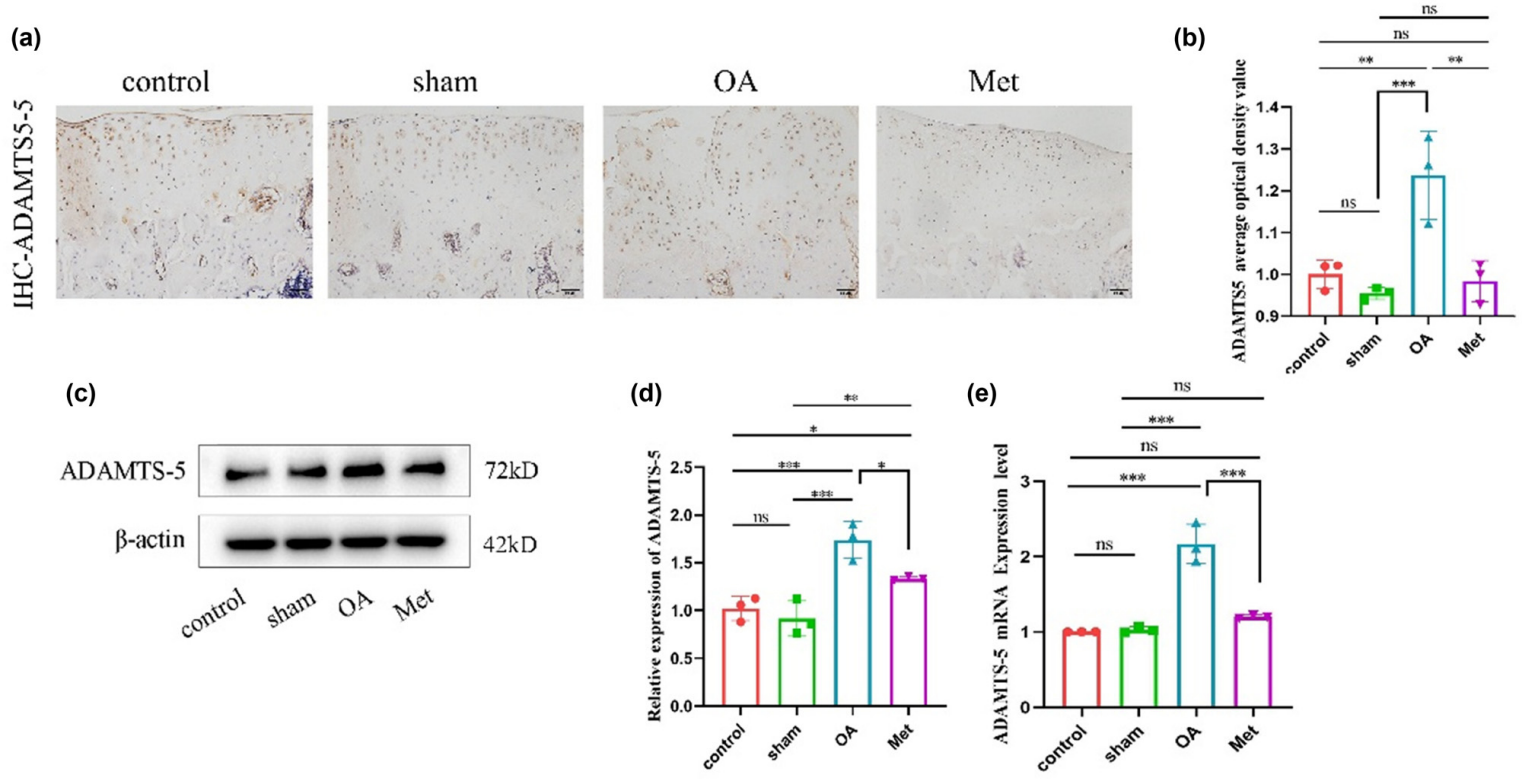
Met inhibits the degradation of cartilage tissues. (a) Immunohistochemistry of ADAMTS5 expression. Scale bar = 50 μm. (b) Quantitative analysis of ADAMTS5 immunohistochemistry. (c) Western blot analysis of ADAMTS5 in cartilage tissue. (d) Quantitative data of ADAMTS5 western blot analysis. Data are presented as mean ± SD (*n* = 3). (e) Measurement of ADAMTS5 mRNA expression by RT-qPCR. Data are presented as mean ± SD (*n* = 3). ns, no significance; *P < 0.05; **P < 0.01; ***P < 0.001.

The balance between synthesis and breakdown metabolism in chondrocytes is essential for ECM homeostasis, as metabolic imbalances have been found to be significant contributors to cartilage degeneration. Thus, we assessed the expression of ADAMTS5 in rat cartilage tissue via immunohistochemistry (Figure 2a). The results indicated that all groups exhibited different levels of ADAMTS5 protein expression, with brownish-yellow particles distributed throughout the cytoplasm. In the sham group, the number of cells positive for ADAMTS5 showed no significant difference compared to the control group. In contrast, the OA group displayed an elevated number of ADAMTS5-positive cells, characterized by more intense staining. Conversely, the Met group demonstrated a decrease in the number of ADAMTS5-positive cells with slightly lighter staining compared to the OA group. The average optical density values for ADAMTS5 positive expression in each group were measured, as shown in Figure 2b. There was no significant difference in ADAMTS5 expression levels between the sham group and the control group (*P* > 0.05). However, the OA group showed an upregulation of ADAMTS5 positive expression compared to both the control and sham groups, while the Met group exhibited a reversal of the surgery-induced upregulation of ADAMTS5 positive expression in rat cartilage tissue (*P* < 0.05).

As shown in Figure 2c and d, the western blot results reveal a significant increase in the expression of ADAMTS5 protein in the OA group. Compared to the OA group, the expression level of ADAMTS5 protein in the Met group was found to significantly decreased (*P* < 0.05), consistent with the results of immunohistochemistry. Additionally, quantitative real-time PCR further validated the results of ADAMTS5 immunohistochemistry and western blot. Moreover, as shown in Figure 2e, the mRNA expression level of ADAMTS5 in the cartilage tissue of the sham group showed no significant difference compared to the control group (*P* > 0.05). Compared to both the control and sham groups, the OA group exhibited increased ADAMTS5 mRNA expression, which could be effectively counteracted by Met in the OA cartilage tissue (*P* < 0.05).

### 3.3 Met promotes the synthetic metabolism of cartilage tissue in rats with OA

**Figure 3:**
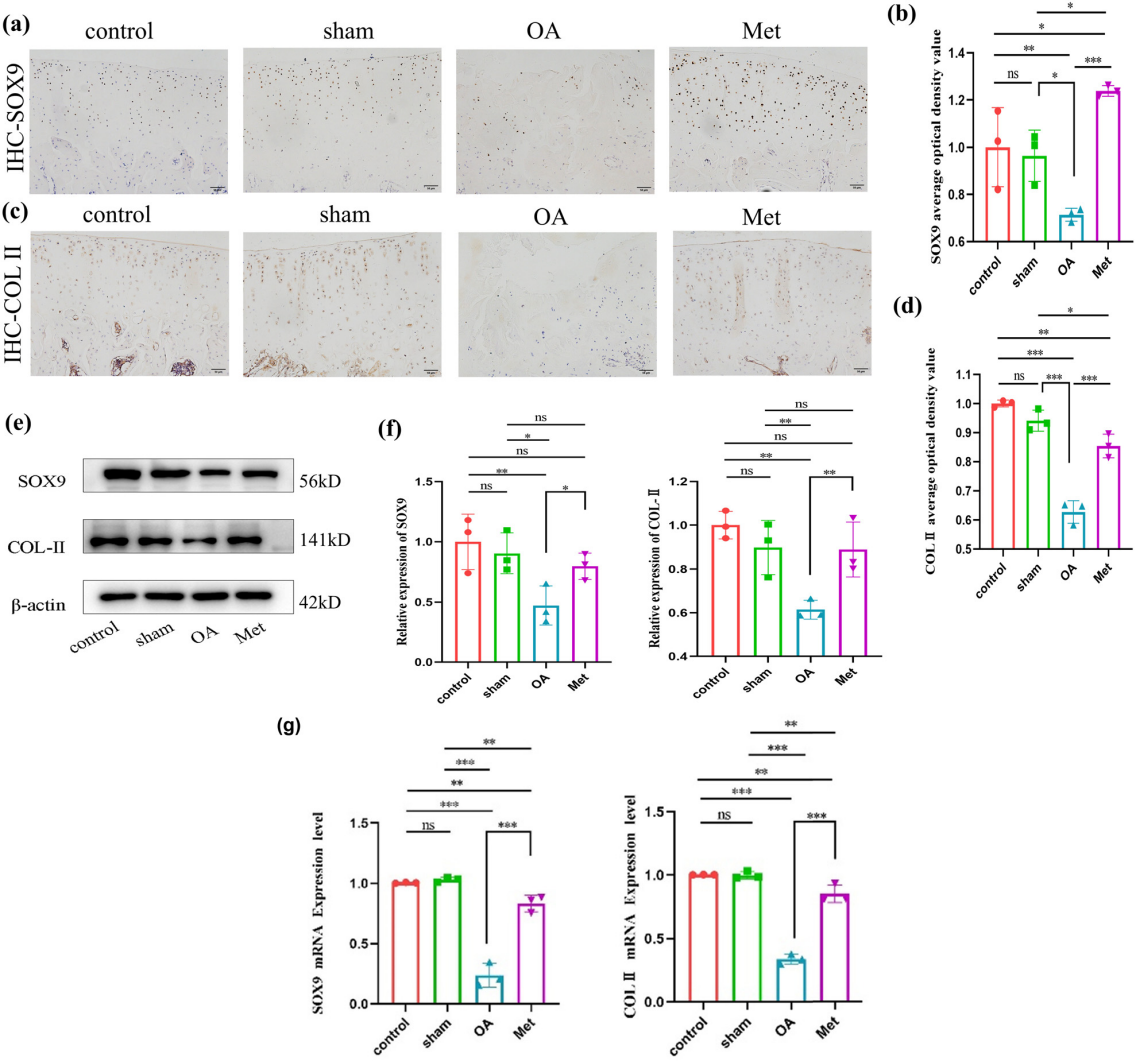
Met promotes cartilage tissue synthesis. (a) Immunohistochemistry of SOX9 expression. Scale bar = 50 μm. (b) Quantitative analysis of SOX9 immunohistochemistry. (c) Immunohistochemistry of COL-II expression. Scale bar = 50 μm. (d) Quantitative analysis of COL-II immunohistochemistry. (e) Western blot analysis of SOX9 and COL-II in cartilage tissue. (f) Quantitative data of SOX9 and COL-II western blot analysis. Data are presented as mean ± SD (*n* = 3). (g) Measurement of SOX9 and COL-II mRNA expression by RT-qPCR analysis. Data are presented as mean ± SD (*n* = 3). ns, no significance; *P < 0.05; **P < 0.01; ***P < 0.001.

One of the factors that promotes the progression of OA is the inhibition of chondrocyte synthesis. Immunohistochemical analysis was conducted to assess the expression of SOX9 and Col-II proteins in the cartilage tissues of each group, and the results reveal varying degrees of expression. Positive expression of SOX9 can be observed as brown-yellow granules distributed in the cell nucleus (Figure 3a), while positive expression of Col-II can be observed as brown-yellow granules distributed in the cytoplasm (Figure 3c). Compared to the control and sham groups, the OA group demonstrated a decrease in the number of SOX9 and Col-II positive cells, resulting in lighter staining. In contrast, the Met group exhibited an increase in the number of SOX9 and Col-II positive cells compared to the OA group, resulting in darker staining. The average optical density values for SOX9 (Figure 3b) and Col-II (Figure 3d) were similar (*P* > 0.05) for the levels of SOX9 and Col-II positive expression in the cartilage tissue of the sham group compared to the control group. However, the OA group demonstrated a significant reduction in the levels of SOX9 and Col-II positive expression compared to the control and sham groups (*P* < 0.05). Notably, the Met group significantly reversed the downregulation of SOX9 and Col-II positive expression in OA cartilage tissue (*P* < 0.05).

The immunohistochemistry results were further confirmed by western blot (Figure 3e and f), which demonstrated that the expression levels of SOX9 and Col-II proteins in the cartilage tissue of the OA group were significantly inhibited compared to the control and sham groups. However, this inhibitory effect could be effectively reversed following Met treatment, leading to the restoration of SOX9 and Col-II protein expression levels (*P* < 0.05). Additionally, quantitative real-time PCR analysis revealed that the mRNA expression levels of SOX9 and Col-II, genes associated with cartilage synthesis, were significantly inhibited in the cartilage tissue of the OA group (*P* < 0.05). Moreover, after being exposed to Met, the previously suppressed mRNA expression levels were found to be effectively reversed (Figure 3g), aligning with the findings from immunohistochemistry and western blot.

### 3.4 Met induces the activation of autophagy in the cartilage tissue of OA rats

**Figure 4:**
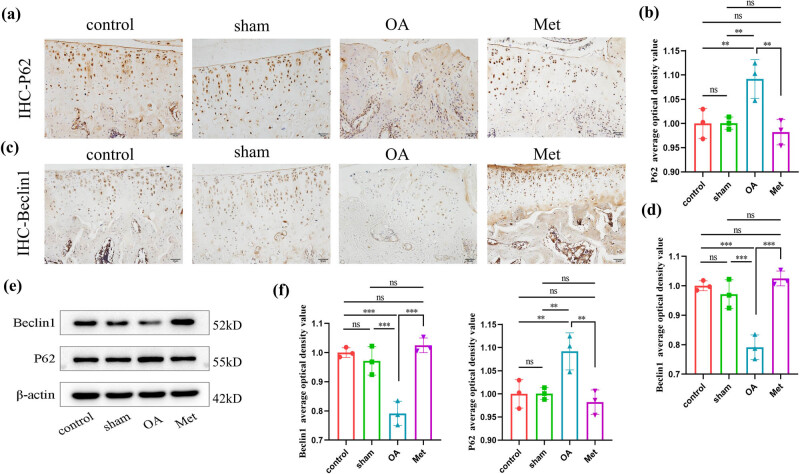
Met inhibits autophagy in chondrocytes. (a) Immunohistochemistry of P62 expression. Scale bar = 50 μm. (b) Quantitative analysis of P62 immunohistochemistry. (c) Immunohistochemistry of Beclin1 expression. Scale bar = 50 μm. (d) Quantitative analysis of Beclin1 immunohistochemistry. (e) Western blot analysis of Beclin1 and P62 in cartilage tissue. (f) Quantitative data of Beclin1 and P62 western blot analysis. Data are presented as mean ± SD (*n* = 3). ns, no significance; *P < 0.05; **P < 0.01; ***P < 0.001.

Autophagy is a regulatory mechanism that maintains cellular homeostasis and protects cartilage. To investigate the impact of Met on autophagy in OA, we measured the levels of Beclin1 and P62 in cartilage tissue as indicators of autophagic activity. As shown in Figure 4a, immunohistochemistry results revealed varying degrees of P62 expression across all groups. The P62 protein was observed as brownish-yellow granules in the cytoplasm. The OA group exhibited an increased number of P62-positive cells with darker staining compared to the control and sham groups. In contrast, the Met group showed a decreased number of P62-positive cells with lighter staining compared to the OA group. As shown in Figure 4c, the Beclin1 protein was also observed as brownish-yellow granules in the cytoplasm. The OA group had a decreased number of Beclin1-positive cells with lighter staining compared to the control and sham groups. Conversely, the Met group exhibited an increased number of Beclin1-positive cells with darker staining compared to the OA group.

Next, we assessed, the average optical density values for immunohistochemical staining (Figure 4b and d). Compared to the control group, we found no statistically significant difference (*P* > 0.05) in the positive expression levels of the autophagy regulatory factor Beclin1 and the autophagy inhibitory factor P62 in the articular cartilage tissue of the sham group. However, in the OA group, the positive expression levels of P62 in the cartilage tissue were significantly higher than those in the control and sham groups (*P* < 0.05). In contrast, the Met group was found to significantly reduce the positive expression levels of P62 in the cartilage tissue compared to the OA group (*P* < 0.05). Additionally, the positive expression levels of Beclin1 in the cartilage tissue of the OA group were significantly lower than those in the control and sham groups (*P* < 0.05), while in the Met group, the positive expression levels of Beclin1 were significantly increased compared to the OA group (*P* < 0.05).

Further experiments demonstrated that in the OA group, the protein expression level of P62 was upregulated, while in the Met group, the protein expression level of P62 was downregulated (*P* < 0.05) (Figure 4e and f). Conversely, the protein expression level of Beclin1 in the cartilage tissue was downregulated in the OA group, whereas it was upregulated in the Met group (*P* < 0.05).

### 3.5 Met effectively inhibits the activation of the PI3K/AKT/mTOR signaling pathway in the cartilage tissue of OA rats

**Figure 5:**
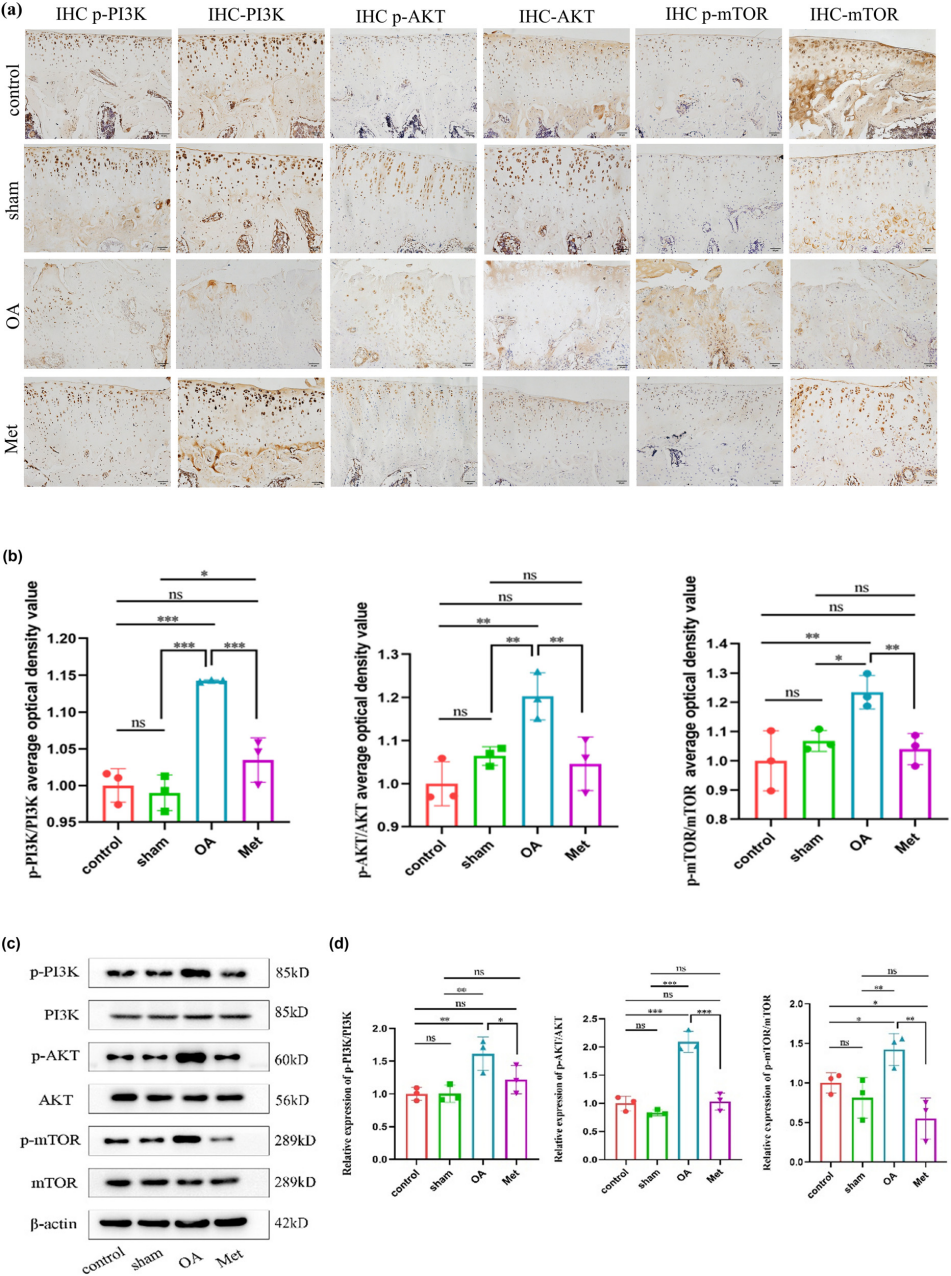
Met inhibits the PI3K/AKT/mTOR signaling pathway activation. (a) Immunohistochemistry of p-PI3K, PI3K, p-AKT, AKT, p-mTOR, and mTOR expression. Scale bar = 50 μm. (b) Quantitative analysis of p-PI3K/PI3K, p-AKT/AKT, and p-mTOR/mTOR immunohistochemistry. (c) Western blot analysis of PI3K, p-PI3K, AKT, p-AKT, mTOR, and p-mTOR in cartilage tissue. (d) Quantitative data of PI3K, p-PI3K, AKT, p-AKT, mTOR, and p-mTOR western blot analysis. Data are presented as mean ± SD (*n* = 3). ns, no significance; *P < 0.05; **P < 0.01; ***P < 0.001.

We further investigated the impact of Met on the PI3K/AKT/mTOR signaling pathway in cartilage tissue. Immunohistochemistry results revealed distinct expressions of p-PI3K, p-AKT, and p-mTOR proteins across the various groups (Figure 5a). Positive staining for these proteins appeared as brownish-yellow granules in the cytoplasm of cells in all groups. The OA group demonstrated an elevated number of positive cells for these factors compared to the control and sham groups. In contrast, the Met group displayed a reduced number of positive cells relative to the OA group, with a lighter staining intensity. The average optical density values of immunohistochemistry were calculated to obtain the relative positive expression levels of p-PI3K/PI3K, p-AKT/AKT, and p-mTOR/mTOR in each group (Figure 5b), and we found no significant difference between the control and the sham groups (*P* > 0.05). In addition, the OA group showed a significant increase in the expression of these proteins compared to the control and sham groups (*P* < 0.05), while the Met group demonstrated a significant decrease compared to the OA group (*P* < 0.05).

Moreover, protein immunoblot analysis showed that the expression levels of p-PI3K, p-AKT, and p-mTOR proteins were highest in the OA group, these protein expression levels were lower in the Met group (Figure 5c). Further analysis of protein band density values revealed an elevation in the expression levels of p-PI3K, p-AKT, and p-mTOR proteins in the cartilage tissue of the OA group, while the expression levels of PI3K, AKT, and mTOR remained relatively stable across all groups (*P* < 0.05), indicating activation of the PI3K/AKT/mTOR signaling pathway in the OA group (Figure 5d). However, Met administration reduced the expression of p-PI3K, p-AKT, and p-mTOR proteins in the cartilage tissue of OA rats. These findings suggest that Met can inhibit the activation of the PI3K/AKT/mTOR signaling pathway in OA cartilage tissue.

## 4 Discussion

OA is a degenerative disease characterized by cartilage degeneration, synovial hyperplasia, osteophyte formation, subchondral bone sclerosis, and the development of subchondral bone cysts. It is a prominent cause of disability within orthopedic conditions [14]. The onset and progression of OA are influenced by a complex interplay of factors, including chondrocyte apoptosis, joint cartilage damage, and other related processes. These components contribute to the gradual deterioration of joint structure and function, ultimately leading to joint pain and inflammatory reactions [15,16].

The current primary focus of OA treatment is symptom management, aiming to reduce pain and swelling. NSAIDs are commonly prescribed to alleviate symptoms [17]. However, these medications have limited effectiveness in improving cartilage degeneration and can cause side effects such as liver damage or gastrointestinal discomfort. Therefore, there is an essential need for alternative medications with fewer side effects and reliable efficacy [18]. Met widely used in the treatment of type 2 diabetes has shown potential therapeutic effects beyond glucose-lowering, including in tumor treatment [19], aging [20], and inflammation [21]. In addition, several studies have demonstrated that Met can inhibit the release of inflammatory mediators, thereby reducing inflammatory reactions [22].

Our present study revealed that administering Met to rats with OA resulted in a significant improvement in cartilage structure compared to the OA group based on the substantial increase in chondrocyte count and a reduction in the loss of proteoglycans, which are essential components of the cartilage matrix. The increased chondrocyte count indicates an enhanced capacity for cartilage repair and regeneration, as these cells are crucial for maintaining the health and functionality of cartilage tissue. Additionally, the decrease in proteoglycan loss points to a reduction in the extent of cartilage degeneration. Taken together, these findings suggest that Met may facilitate the process of cartilage repair and regeneration.

The primary factor causing cartilage degeneration is the metabolic imbalance between cartilage synthesis factors and degradation factors. During cartilage degradation, ADAMTS5 plays an important role in cleaving proteoglycans, leading to cartilage damage [23]. The expression level of ADAMTS5 in cartilage tissue is closely associated with the severity of joint cartilage damage, with its higher expression levels correlating with more severe joint damage [24]. In our present study, upregulation of ADAMTS5 expression was observed in the OA group, and after treatment with Met, we observed a significant decrease in ADAMTS5 expression. Additionally, we evaluated the expression levels of markers associated with synthetic metabolism, specifically SOX9 and COL-II, in cartilage tissue. In the OA group, these factors were downregulated but could be reversed following Met treatment. These results further support the reduction of cartilage damage after Met treatment consistent with previous research findings [25].

Recent studies have highlighted the association between OA progression and alterations in autophagy within chondrocytes [26]. Autophagy is a highly conserved cellular metabolic process that clears harmful substances from cells and maintains a dynamically stable intracellular environment. This process enhances cellular metabolic activity, slowing the progression of OA, and is considered an important protective mechanism in OA [27]. Autophagy activity can be modulated by changes in regulatory factors; increased autophagy activity leads to upregulation of positive regulatory factors and downregulation of negative regulatory factors, thereby maintaining intracellular autophagy stability. However, during the development of OA, chondrocytes undergo increased abnormal metabolism, resulting in decreased cellular autophagy activity [28]. The impaired regulation of the autophagic process can cause dysfunction or death of chondrocytes, leading to abnormal synthesis and degradation metabolism, ECM degradation, and the occurrence of OA [29]. Beclin1 facilitates the formation of autophagosomes and initiates autophagy, and reduced expression of Beclin1 has been found to decrease the number or functionality of autophagosomes, thereby impairing the initiation of the autophagy pathway [30]. Conversely, P62 is involved in the aggregation, degradation, and clearance of abnormal proteins during autophagy, and its expression level is negatively correlated with cellular autophagy [31]. Thus, by activating Beclin1 and inhibiting the expression of P62 in chondrocytes, it is possible to improve abnormal autophagy activity and alleviate the progression of OA. Therefore, we investigated the protective effect of Met treatment on chondrocyte autophagy. The findings showed decreased autophagy levels and increased ADAMTS5 expression in cartilage tissues in the OA group, indicating suppressed autophagic activity in chondrocytes during OA development, leading to ADAMTS5 accumulation on exacerbating chondrocyte degradation metabolism. Conversely, in the Met-treated rat model, lower levels of P62 and ADAMTS5 proteins, along with higher levels of Beclin1 protein, were observed, suggesting that Met treatment reversed autophagy inhibition, facilitating the clearance of accumulated ADAMTS5 in cartilage tissues. Thus, it can be deduced that Met might be effective in enhancing chondrocyte autophagic function, restoring the balance between chondrocyte synthesis and degradation metabolism, and ultimately delaying the progression of OA.

The PI3K/AKT/mTOR signaling pathway is a complex and essential mechanism for cellular signal transduction, regulating fundamental cellular functions such as the cell cycle, metabolism, and proliferation. This pathway is essential for maintaining the balance and stability of the internal environment of the organism [32]. There are pieces of evidence suggesting that the PI3K/AKT signaling pathway plays a significant role in the pathological process of OA. Previous studies have shown that inhibiting the activation of the PI3K/AKT pathway can improve excessive production of ROS induced by IL-1β and chondrocyte apoptosis [33]. As a downstream target of the PI3K/AKT pathway, mTOR regulates autophagy, and its abnormal expression may lead to dysregulation of autophagic function, thereby affecting cell survival and metabolism [34]. Inhibiting the promotion of autophagy by the PI3K/AKT/mTOR signaling pathway has been reported as an effective strategy to alleviate the progression of OA [35,36].

Moreover, we investigated whether the enhanced autophagy induced by Met in chondrocytes might be related to the inhibition of the PI3K/AKT/mTOR signaling pathway. In the OA rat model, we found an elevation in the phosphorylation levels of PI3K, AKT, and mTOR in cartilage tissues, as well as suppressed autophagic activity in chondrocytes, indicating that excessive activation of the PI3K/AKT/mTOR signaling pathway disrupted the autophagic process, leading to an imbalance in chondrocyte function and homeostasis. In addition, Met was found to reduce the activation levels of PI3K, AKT, and mTOR in cartilage tissues while promoting Beclin1 expression and inhibiting P62 levels. Additionally, Met treatment suppressed ADAMTS5 production and promoted the expression of SOX9 and Col-II. These indicate that inhibiting the activation of the PI3K/AKT/mTOR pathway can improve cell autophagy and promote chondrocyte synthesis metabolism and that Met can enhance chondrocyte autophagy by inhibiting the abnormal activation of the PI3K/AKT/mTOR pathway, reducing cartilage matrix degradation, and thus improving cartilage injury.

In conclusion, the results of this study indicate that Met can inhibit the activation of the PI3K/AKT/mTOR pathway, enhance autophagy activity in chondrocytes, and reduce the degradation of the cartilage matrix, which effectively slows down the progression of cartilage degeneration, alleviates symptoms of OA, and provides protective effects on articular cartilage in individuals with OA.
